# Supplementary material for: Concussion-Related Biomarker Variations in Retired Rugby Players and Implications for Neurodegenerative Disease Risk: The UK Rugby Health Study
Source: Int J Mol Sci. 2024 Jul 17;25(14):7811. doi: 10.3390/ijms25147811 (PMC11276902; doi:10.3390/ijms25147811)
Supplement: Supplementary file 1 [file ijms-25-07811-s001.zip › ijms-3078067-supplementary.pdf]

Supplementary Figure S1

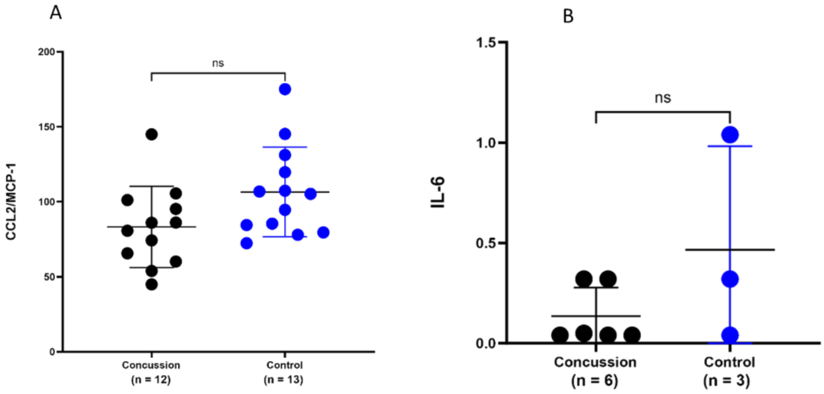

Figure S1: A) CCL2/MCP-1. Not statistically different *P*= 0.068. B) IL-6. Not statistically different *P*= 0.381 on the Mann-Whitney U test.
